# Television exposure and overweight/obesity among women in Ghana

**DOI:** 10.1186/s40608-018-0186-4

**Published:** 2018-02-14

**Authors:** Derek Anamaale Tuoyire

**Affiliations:** Department of Community Medicine, School of Medical Sciences, College of Health and Allied Sciences, University of Cape Coast, Cape Coast, Ghana

**Keywords:** Television exposure, Obesity, Overweight, Women, Ghana

## Abstract

**Background:**

Although the public health importance of the association between television (TV) viewing and obesity and/or related outcomes have been demonstrated in both cross-sectional and prospective studies elsewhere, similar studies are lacking within the African region. With the view to fill this gap in the literature, the current study explored the association between TV exposure and overweight/obesity among Ghanaian women.

**Methods:**

Based on a sample of 4158 women, descriptive statistics and binary logistic regression were applied to data on TV ownership, TV viewing frequency, and body mass index (BMI) measures from the 2014 Ghana Demographic and Health Survey (GDHS) to explore the association between TV exposure and overweight/obesity among Ghanaian women.

**Results:**

Despite controlling for other factors (age educational level, marital status, wealth quintile, occupation, type of locality, and parity), the results show that women with TV in their households, and with high TV exposure were significantly (*P* < 0.05) more likely (OR = 1.39, 95% CI = 1.002, 1.923) to be overweight/obese compared to those with no TV in their households, and no TV exposure.

**Conclusion:**

The study demonstrates that increased TV exposure is significantly associated with overweight/obesity among women in Ghana even after adjusting for other factors. Interventions aimed at tackling obesity in Ghana should focus on encouraging the uptake of more physically demanding pastime activities in place of TV “sit time”.

## Background

There is growing concern about the rising global prevalence of overweight and obesity, as well as their associated adverse health implications [1–3]. One feature of this global problem is the fast pace at which developing countries are being affected compared to the developed countries. For example, while the number of people affected by overweight or obesity increased 1.7 times between 1980 and 2008 in developed countries, those affected in developing countries more than tripled from around 250 million people to 904 million over the same period [4].

As in many developing countries, the prevalence of overweight and obesity in Ghana has consistently increased over the last few decades, disproportionately affecting women than men. For instance, the Ghana Demographic and Health Survey (GDHS) estimated the prevalence of overweight or obesity among women (40%) aged 15–49 years to be more than twice that of men (16%) [5]. Specifically, the prevalence among women was estimated to have increased by 27% between 1993 and 2014 [5]. Further, the prevalence of overweight or obesity in Ghana affects more urban than rural residents, and increases with household wealth and level of education [5].

Although overweight and obesity result from energy imbalance (consuming more calories than are equivalently expended in physical activity), studies have found a variety of factors to be associated with the phenomenon. Various associations, in terms of magnitude and direction have been reported between overweight and obesity, and a number of factors including age [6, 7], gender [8, 9], socioeconomic status [10, 11], marital status [12, 13], education [14, 15], occupation [6, 15], ethnicity [6, 14], genetics [16], dietary [17, 18] and physical activity patterns [19]. For instance, contrary to developed countries, the wealthier, more educated and urban populations are more at risk of overweight and obesity in developing countries [10, 11, 20]. Prior studies [6, 21, 22] in Ghana have found overweight and obesity to be positively associated with  age, household wealth, education, being married, and parity among others.

Engaging in sedentary behaviours, such as television (TV) viewing has been implicated among the multiplicity of the factors underlying the increasing prevalence of overweight and obesity observed in many populations around the world [23, 24]. A number of theoretical propositions about the possible mechanisms through which TV viewing affects overweight and obesity have been advanced. It is hypothesized that, TV viewing displaces participation in high-intensity discretionary physical activity, reduces resting energy expenditure compared to other activities, and increases sleep deprivation [19, 25]. Nonetheless, studies exploring this hypothesis have found a weak relationship between TV viewing and physical activity [19, 25].

An alternatively hypothesis posited is that TV viewing leads to an increase in overall energy intake either indirectly through exposure to advertising and consequent intake of foods commonly advertised on TV, or directly through the consumption of high calorie foods and beverages while viewing TV [17, 18]. Indeed studies involving both children and adults have found a positive association between TV viewing and intake of high calorie foods such as soda, pizza, and high-energy snacks [17, 18, 23]. In an experimental study involving children, Blass et al. [26] found children’s energy intake to be higher when viewing TV than in control conditions when the TV is switched off.

Beyond exploring the possible mechanisms between TV viewing and physical activity or calorie intake, positive associations between TV viewing and obesity and/or related indicators have consistently been observed across space and time. Studies in the United States (US) have found that men and women viewing the most TV have an increased risk of obesity compared with those viewing the least TV [27–29]. In Australia, women who viewed TV for more than three hours a day were found to have a higher prevalence of severe abdominal obesity; while men who viewed TV for the same amount of time had a higher prevalence of moderate abdominal obesity [23]. More recently, a worldwide cross-sectional study across low, middle, and high income countries involving 207,672 adolescents from 37 countries and 77,003 children from 18 countries found increased TV viewing hours to be positively associated with body mass index (BMI) [30].

Although the public health importance of the association between TV viewing and obesity or related outcomes have been demonstrated in both cross-sectional and prospective studies involving children, adolescent and adult samples in other regions of the world, similar studies are rare within the African region. Nonetheless, while examining the relationship between ownership of different types of household assets and BMI among Ghanaian women, Dake and Fuseini [31] tangentially observed that women who reported viewing TV almost every day were likely to have obesity compared to those who did not. Taking cue from Dake and Fuseini [31], this study explored the association between TV exposure and overweight/obesity in Ghana with the view to contributing to the discourse on TV viewing and obesity, particularly in the African region.

In Ghana, TV ownership has been increasing since the inception of Television broadcasting in 1965. The sixth Ghana Living Standard Survey (GLSS) [32] estimated TV ownership to have increased between 1998/99 and 2012/13 from 40 to 75% and from 12 to 34% in urban and rural households, respectively. Indeed, hardly would one pass by a street, shop, bar, restaurant, bus station or even an office in most cities, towns and villages in Ghana without seeing a TV streaming. The screening of foreign telenovelas (soap operas) during primetime has become a means for competing stations to attract large audience, particularly women [33]. Telenovelas seem to have gone “viral” to the extent that TV stations screen them almost simultaneously with people – mostly women – seated and viewing for considerable hours each day. Some TV stations translate telenovelas into the local language (Twi) which makes it the more attractive for those who would otherwise have been deterred by language barriers.

With TV viewing considerably a part of life in Ghana today, this study explored the relationship between TV viewing and overweight/obesity among Ghanaian women using data from the 2014 GDHS. The study focused on women mainly because of the disproportionately high and rapid rates of overweight/obesity among Ghanaian women, compared to men [5]. In addition, Ghanaian women have also been found to report low levels of vigorous physical activity, which could increase their risk of developing overweight/obesity [34, 35]. This study would be useful in broadening our understanding of some of the drivers of obesity in Ghana or Africa, as well as at-risk groups for designing interventions.

## Methods

### Data source

The data used for the current study is the 2014 GDHS by Ghana Statistical Service (GSS), Ghana Health Service (GHS), and Inner City Fund (ICF) International. Specifically, the women’s dataset was used given that they constituted the population of interest in this study. The GDHS collected the data using a two-staged stratified random sampling procedure. At the initial stage, clusters were selected using systematic random sampling. The sampling frame for selection of the clusters was an updated list of enumeration areas used in the 2010 Ghana Population and Housing Census. This was then followed with the selection of households in each cluster through a systematic random sampling procedure. Eligible women who provided signed written consent to participate in the survey were interviewed.

The GDHS collected anthropometric measures of all eligible women. To this end, a SECA 874 digital scale was used to measure their weight in 0.01 kg, while a Shorr height board was used to measure their height to the nearest 0.1 cm. Additional information regarding protocols used for taking these anthropometric measurements can be found in the MEASURE DHS Biomaker field manual [36]. The total sample for this study, excluding all pregnant women and lactating mothers was 4158. Permission was obtained from MEASURE DHS to download the 2014 GDHS raw data from http://dhsprogram.com/data/available-datasets.cfm for the purpose of the current study.

### Study variables

The outcome variable in this study was generated from BMI scores of the women. A binary outcome variable was generated guided by the standard World Health Organisation’s (WHO) BMI cut-off points (underweight, < 18.5 kg/m^2^; normal weight, 18.5–25 kg/m^2^; overweight, 25.0–29.9 kg/m^2^; obese, ≥ 30.0 kg/m^2^). Hence, for the purpose of this study and consistent with prior studies [5, 6, 21, 37], a BMI of 25.0 kg/m^2^ or more was categorised as overweight/obese while a BMI lower than 25.0 kg/m^2^ was categorised otherwise.

The key independent variable for this study was generated from two variables. The first variable assessed whether there was a TV in the household of respondents, with two response options: yes and no. The second variable assessed respondent’s frequency of viewing TV, with three response options: not at all, less than once a week (moderate), and at least once a week (high). For the purpose of this study, not at all, less than once a week, and at least once a week were considered as: no exposure, moderate exposure, and high exposure, respectively. Based on these two variables, another categorical variable (TV exposure) was generated out of possible outcomes of the presence of TV in a household and the frequency of TV viewing and coded with the following options: “no TV in household, no TV exposure” = 0; “no TV in household, moderate TV exposure” = 1; “no TV in household, high TV exposure” = 2; “TV in household, no TV exposure” = 3; “TV in household, moderate TV exposure” = 4; and “TV in household, high TV exposure” = 5.

**Fig. 1:**
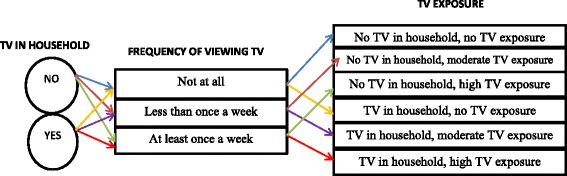
Schematic of TV exposure classification

This categorisation of TV exposure as presented in Fig. 1 is meant to demonstrate the scenarios of TV exposure in Ghana. In Ghana, it is typical for people who do not have TV sets in their household to spend time in neighbouring homes or nearby shops viewing TV. Communal TV viewing is also a common phenomenon in Ghana – where those who own TV sets take them outdoors for other community members to join in viewing. Any of these forms constitutes a certain level of TV exposure with possible implications on physical activity, calorie intake and general health of the involved.

Other explanatory variables in this study included age (15–24, 25–34, 40–44, and 45+); educational level (no education, primary, middle/junior secondary school (JSS)/junior high school (JHS), and secondary/higher); marital status (never married, married, cohabiting, and (divorced/widowed/separated); occupation (not working, professional/managerial, sales/trade, agricultural and manual labour); wealth quintile (poorest, poorer, middle, rich and richest); type of locality (rural, and urban); and parity (zero, one, two, three, four, five, six, and seven or more). Parity was included as proxy to account for the potential effect of childbearing on women’s weight as noted in the literature [38, 39].

### Statistical analysis

Both descriptive and inferential statistics were applied in estimating the association between TV exposure and overweight/obesity using STATA 11.0 software. The descriptive analysis used percentages to estimate the prevalence of TV exposure and overweight/obesity in relation to the characteristics of women in the study (Table 1). In the second level of analysis, two binary logistic regression models were conducted to estimate the effect of TV exposure on overweight/obesity. In this regard, there was a bivariate model (Model I) consisting of only TV exposure, and a multivariate model (Model II) in which the other aforementioned explanatory variables were included. The GDHS survey design and weighting factors were taken into consideration in all analyses. To test for fitness of the models, the Hosmer- Lemeshow goodness-of-fit test was used.

**Table 1:** Television exposure and overweight/obesity prevalence by background characteristics of women in Ghana, GDHS: 2014

	No TV in household, no TV exposure (*n* = 824)	No TV in household, moderate TV exposure (*n* = 367)	No TV in household, high TV exposure (*n* = 345)	TV in household, no TV exposure (*n* = 183)	TV in household, moderate TV exposure (*n* = 693)	TV in household, high TV exposure (*n* = 1746)	Overweight/obesity	Total
Characteristic	%	%	%	%	%	%	%	No.
Age group (years)								
15–24	19.0	10.3	11.1	5.0	14.4	40.3	17.6	1445
25–34	17.4	7.6	7.3	3.0	17.9	46.8	48.4	1242
35–44	21.4	8.8	6.1	4.9	17.9	41.0	56.2	1062
45+	25.9	7.8	6.8	5.7	17.7	36.0	52.6	410
Total	19.8	8.8	8.3	4.4	16.7	42.0	40.1	4158
Educational level								
No education	44.2	10.3	7.6	5.1	11.7	21.1	26.8	794
Primary	24.1	14.3	9.5	4.6	13.4	34.0	37.4	765
Middle/JHS	14.3	8.2	9.6	4.7	20.0	43.1	42.8	1710
Secondary/higher	4.8	4.0	5.4	3.0	17.5	65.4	49.2	889
Total	19.8	8.8	8.3	4.4	16.7	42.0	40.1	4158
Marital status								
Never married	16.1	9.7	11.1	4.5	15.8	42.8	22.1	1408
Married	22.6	6.8	4.4	4.5	18.3	43.4	50.1	1699
Cohabiting	19.1	9.4	7.5	4.8	13.8	45.4	40.8	585
Wid/div/sep	21.5	12.9	15.0	3.2	17.1	30.2	57.4	466
Total	19.8	8.8	8.3	4.4	16.7	42.0	40.1	4158
Wealth quintile								
Poorest	62.9	12.1	8.7	3.6	4.9	7.8	12.6	696
Poorer	37.3	19.0	14.6	4.1	8.6	16.4	24.8	709
Middle	11.5	13.0	15.8	7.1	18.0	34.6	38.6	863
Richer	1.3	3.6	3.8	4.5	24.5	62.3	52.5	925
Richest	1.0	0.2	1.0	2.7	22.4	72.6	60.6	967
Total	19.8	8.8	8.3	4.4	16.7	42.0	40.1	4158
Occupation								
Not working	18.6	9.4	8.7	4.8	14.8	43.8	23.8	968
Prof/managl/cleric	3.9	3.6	5.3	2.0	22.5	62.8	58.6	348
Sales/trade	10.9	7.0	7.5	4.7	20.7	49.2	54.6	1535
Agric	47.3	13.1	9.7	3.9	8.8	17.2	19.7	780
Manual	18.0	10.6	9.8	5.3	16.1	40.1	45.7	523
Total	19.8	8.8	8.3	4.4	16.7	41.9	40.1	4153
Type of locality								
Urban	6.0	5.6	7.4	4.1	18.5	58.3	49.2	2289
Rural	36.7	12.8	9.3	4.8	14.4	22.0	28.9	1869
Total	19.8	8.8	8.3	4.4	16.7	42.0	40.1	4158
Parity								
Zero	15.4	9.0	10.2	4.3	15.0	46.0	23.7	1327
One	12.3	9.3	10.7	4.0	16.9	46.9	39.2	531
Two	15.0	6.6	7.5	2.7	18.8	49.3	55.1	567
Three	18.5	8.4	7.3	4.2	18.3	43.3	55.4	495
Four	21.4	9.8	3.8	6.1	18.8	40.1	50.6	405
Five	28.0	6.3	8.2	5.2	22.5	29.8	43.1	319
Six	28.9	14.9	6.9	6.0	12.6	30.7	49.8	237
Seven or more	47.6	8.5	6.2	4.5	10.6	22.6	35.4	278
Total	19.8	8.8	8.3	4.4	16.7	42.0	40.1	4158
TV exposure								
No TV in household, no TV exposure							20.7	824
No TV in household, moderate TV exposure							25.9	367
No TV in household, high TV exposure							29.2	345
TV in household, no TV exposure							30.3	183
TV in household, moderate TV exposure							50.7	694
TV in household, high TV exposure							51.2	1746
Total							40.1	4158

## Results

**Fig. 2:**
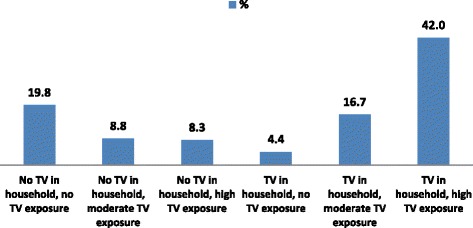
Percentage distribution of TV exposure classification

Table 1 shows the distribution of background characteristics of women in relation to TV exposure and prevalence of overweight/obesity. In general, TV exposure ranged from about 4% among those with TV in their households, but with no TV exposure to 42% among those with TV in their households, and with high TV exposure. This is further illustrated in Fig. 2. TV exposure varied across the various age cohorts considered in this study. For example, while slightly more than a quarter (26%) of those with no TV in their households, and no TV exposure were 45 years or older, 47% of those with TV in their households, and with high TV exposure were within the 25–35 year cohort.

TV exposure also varied by educational status. The proportion of those with no TV in their households, and with no TV exposure decreased as level of education increased (from about 44% among those with no education to approximately 5% among those with secondary/higher education). In contrast, the proportion of those with TV in their households, and with high TV exposure seemed to increase with each higher level of educational. This ranged from about 21% among those without education to about 65% among those with secondary/higher education. The results on marital status show that 15% of women without TV in their households, but with high TV exposure were in the widowed/divorced/separated category; while approximately 45% of their counterparts with TV in their households and with high TV exposure were cohabiting.

More than two-thirds of those in the poorest wealth quintile had no TV in their households, and no TV exposure. However, the proportion of women with TV in their households, and with high TV exposure increased along with wealth quintile from about 8% in the poorest quintile to about 73% in the richest quintile. With regards to occupation, a greater proportion of those with TV in their households, and with moderate TV exposure (23%), and those with TV in their households, and with high TV exposure (63%) were engaged in professional/managerial/clerical occupations. The results also show differences in TV exposure by type of locality. For instance, while more rural (9%) than urban (7%) women had no TV in their households, but had high TV exposure; more urban (58%) than rural (22%) women had TV in their households, and had high TV exposure.

The prevalence of overweight/obesity among the women in this study was 40%, with some variations observed in relation to their TV exposure and background characteristics (Table 1). Overweight/obesity ranged from about 21% among those with no TV in their households, and no TV exposure to about 51% among those with TV in their households, and with high TV exposure. Regarding the other characteristics of women, the prevalence of overweight/obesity increased with age, educational level, and wealth quintile. Overweight/obesity was highest among widowed/divorced/separated (57%) women, those in professional/managerial/clerical (59%) occupations, urban (49%) dwellers, and those with three children (55%).

The logistic regression results on the association between TV exposure and overweight/obesity is presented in Table 2. The results in Model I show that the likelihood of developing overweight/obesity was significantly higher among women across all the categories of TV exposure, except for those with no TV in their households, but with moderate TV exposure. Specifically, women who had TV in their households, and with high TV exposure had the highest likelihood (OR = 4.01, 95% CI = 3.214, 4.999) of overweight/obesity compared with the other categories of TV exposure.

**Table 2:** Logistic regression results on television exposure and overweight/obesity among women in Ghana, GDHS: 2014

	Model I		Model II	
Characteristic	OR	95% CI	OR	95% CI
TV exposure				
No TV in household, no TV exposure				
No TV in household, moderate TV exposure	1.338	[0.963,1.860]	0.987	[0.670,1.454]
No TV in household, high TV exposure	1.578^**^	[1.130,2.203]	1.219	[0.833,1.784]
TV in household, no TV exposure	1.662^*^	[1.088,2.537]	0.751	[0.436,1.293]
TV in household, moderate TV exposure	3.933^**^	[3.004,5.151]	1.364	[0.956,1.946]
TV in household, high TV exposure	4.008^**^	[3.214,4.999]	1.388^*^	[1.002,1.923]
Age group (years)				
15–24				
25–34			2.447^**^	[1.819,3.294]
35–44			3.790^**^	[2.642,5.436]
45+			3.568^**^	[2.312,5.505]
Educational level				
No education				
Primary			1.493^**^	[1.122,1.985]
Middle/JHS			1.489^**^	[1.133,1.958]
Secondary/higher			2.045^**^	[1.412,2.962]
Marital status				
Never married				
Married			1.746^**^	[1.211,2.519]
Cohabiting			1.365	[0.929,2.006]
Wid/div/sep			2.033^**^	[1.335,3.097]
Wealth quintile				
Poorest				
Poorer			1.914^**^	[1.386,2.643]
Middle			3.087^**^	[2.143,4.447]
Richer			4.948^**^	[3.212,7.622]
Richest			7.124^**^	[4.437,11.44]
Occupation				
Not working				
Prof/managl/cleric			1.560^*^	[1.063,2.290]
Sales/trade			1.447^**^	[1.120,1.870]
Agriculture			0.610^**^	[0.437,0.852]
Manual			1.459^*^	[1.059,2.010]
Type of locality				
Urban				
Rural			1.335^*^	[1.048,1.702]
Parity				
Zero				
One			1.169	[0.807,1.692]
Two			1.817^**^	[1.205,2.741]
Three			1.791^*^	[1.138,2.818]
Four			1.317	[0.820,2.114]
Five			1.275	[0.778,2.090]
Six			2.156^**^	[1.260,3.690]
Seven or more			1.807^*^	[1.053,3.099]
Wald ×^2^	210.81 (5) 0.0000 0.000 (4) 1.0000		652.95 (30) 0.0000 4.53 (8) 0.8068	
Prob. > x^2^				
Hosmer-Lemeshow X^2^ (d.f)				
Prob. > x^2^				

*OR* Odds Ratios; 95% confidence intervals in brackets

* *p* < .05, ** *p* < .01

From the multivariate results (Model II), however, it was observed that only those with TV in their households, and with high TV exposure were significantly more likely (OR = 1.39, 95% CI = 1.002, 1.923) to have overweight/obesity, compared to those with no TV in their households, and no TV exposure. With the exception of marital status and parity, significant associations were observed between overweight/obesity and all the other background characteristics included in the multivariate model.

## Discussion

This study sought to explore the association between TV exposure and overweight/obesity among Ghanaian women. Despite the attenuation of the association between TV exposure and overweight/obesity after adjusting for other factors, the results show that women who had TV in their households, and had high TV exposure were significantly more likely to have developed overweight/obesity than their counterparts with no TV in their households, and no TV exposure. This finding resonates with previous studies [23, 30, 31, 40] on the association between TV viewing and obesity, irrespective of the fact that such studies have reported varied magnitudes of association, depending on the covariates studied and how TV viewing and obesity were measured and categorized.

The cross-sectional nature of this study limits the possibility of drawing direct cause-and-effect conclusions based on the results. Nonetheless, a number of plausible explanations for the findings could be surmised, particularly in the light of existing body of knowledge on the subject. First, it is possible that women who have TV in their households, inherently spend a lot of time viewing TV in place of engaging in exercise or other forms of rigorous physical activities (for example, traditional dancing or wrestling competitions among others), resulting in an overall decrease in energy expenditure and the subsequent development of overweight/obesity [17, 18, 23, 40].

There is evidence of reduced physical activity globally, which is attributable to gradual shifts from outdoor leisure-time physical activities to sedentary types of activity such as viewing television [41]. In the developing world for instance, Ng and Popkin [42] report high declines in physical activity and a shift towards sedentary activity in domestic domains aided by greater access to home appliances including TV and computers. Hence, it might as well be the case that women with overweight/obesity resort to TV viewing as their main form of recreation rather than engaging in more physically demanding recreational activities such as sports.

An alternative explanation is that the more time such women spend viewing TV, the more they are likely to be inundated with the dozens of commercials and programmes which promote the consumption of high calorie foods [17, 18, 43]. Indeed, commercials on food and beverages (both alcoholic and non-alcoholic) seem to dominate in all the TV stations available in Ghana. Besides the potential of TV commercials to influence dietary patterns and risk of overweight/obesity among Ghanaian women, there is equally the tendency that such women have the habit of consuming high calorie food or drinks simultaneously while viewing TV without realising the quantities being consumed [17, 18].

Overall, a cycle of mutual interaction and reinforcement between TV viewing, physical activity, and dietary patterns could be at play [41], leading to an increased risk of overweight/obesity among Ghanaian women. Thus, as more time is spent viewing TV, less time is spent engaging in physical activities, while the consumption of high caloric food increases, leading to the development of overweight/obesity. And with the onset of overweight/obesity, the drive to substitute more active pastime activities for passive ones such as TV viewing increases, and the cycles continues on.

The positive effect of TV exposure on overweight/obesity could equally be a function of other confounders as prior studies [17, 18] have noted. Indeed the strong association found between the control variables in the present study and overweight/obesity, and their effect on the link between TV exposure and overweight/obesity demonstrate this possibility. In particular, the strong positive effect of education and wealth on overweight/obesity in this study highlight the consistent effect of socioeconomic status on the risk of developing overweight/obesity typically observed in developing countries such as Ghana [6, 22], in contrast to developed countries [10, 11]. In developing countries, including Ghana, socioeconomic development has been found to be associated with changes in dietary and physical activity patterns such that Western type foods and energy saving devices replace traditional (healthier) staple foods and ways of doing things [31, 44].

Apart from the socioeconomic dimensions which seem to be playing out in this study, socio-cultural interpretations are equally plausible. Some Ghanaian women might still hold on to the historic Ghanaian perceptions that large-bodied women are beautiful, healthier and more prestigious, and may therefore be engaging in fattening dietary and physical activity patterns [45, 46]. Contrary to the reported protection that residing in a rural locality provides against overweight/obesity, this study found women in rural localities to be at risk of developing overweight/obesity. As Tuoyire et al. [6] noted, this could be the result of the gradual exposure of rural residents to obesogenic risks commonly seen in urban areas.

Much as the study provides some useful insights for interventions to remedy the problem of overweight/obesity in Ghana, the results need to be interpreted with caution in the light of some limitations. As already alluded to in the preceding section, the study cannot directly establish cause-and-effect relationship between TV exposure and overweight/obesity due to the fact that the data was collected based on a cross-sectional design. Nonetheless, the associations established in this study are to a large extent consistent with prior prospective [27] and cross-sectional studies [23, 30] that have demonstrated connections between TV exposure/viewing/watching and the development of overweight/obesity. Secondly, due to lack of data, the study could not control for other potential mediators of the relationship between TV exposure and overweight/obesity, most importantly dietary behaviour, physical activity and genetic factors. In a related development, the measurement of TV exposure as used in this study could not account for specific duration of TV viewing; neither could it account for the content of the messages to which study participants were exposed. Perhaps, the temporal amount and content of exposure to TV messages is one research area that needs to be explored further.

These limitations notwithstanding, the current study is about the first to specifically examine the relationship between TV exposure and overweight/obesity in Ghana and Africa where overweight/obesity and associated non-communicable diseases are increasing rather rapidly. An additional strength of this study is that the findings can be generalised to a large extent, since national level GDHS data with a high response rate (98.5%) was used. Further, the study contributes to the TV-obesity discourse and broadens our knowledge on some of the potential drivers of overweight/obesity in Ghana and perhaps the rest of the African region.

## Conclusion

The study demonstrates that increased TV exposure is significantly associated with overweight/obesity among women in Ghana even after adjusting for other factors. Interventions aimed at tackling obesity in Ghana should focus on encouraging the uptake of more physically demanding pastime activities (such as brisk-walking and dancing) in place of TV “sit time”. More importantly, intervention planners should consider using the TV as an important means to channel tailored behavioural change communication messages that seek to promote healthy dietary and physical activity behaviours.

## Abbreviations

BMI: Body Mass Index
DHS: Demographic and Health Survey
GDHS: Ghana Demographic and Health Survey
GHS: Ghana Health Service
GSS: Ghana Statistical Service
ICF: Inner City Fund
JHS: Junior High School
JSS: Junior Secondary School
WHO: World Health Organization
